# Prediction of T staging in PI-RADS 4–5 prostate cancer by combination of multiparametric MRI and ^68^Ga-PSMA-11 PET/CT

**DOI:** 10.1186/s12894-023-01376-6

**Published:** 2023-12-11

**Authors:** Yuanzhen Ding, Chenghao Mo, Qiubo Ding, Tingsheng Lin, Jie Gao, Mengxia Chen, Wenfeng Lu, Jiyuan Sun, Feng Wang, Shiming Zang, Qing Zhang, Shiwei Zhang, Hongqian Guo

**Affiliations:** 1 Department of Urology, Nanjing Drum Tower Hospital, Affiliated Hospital of Medical School, Nanjing University, 321 Zhongshan Road, Nanjing, 210008 Jiangsu China; 2 Department of Urology, Drum Tower Hospital Clinical College of Nanjing Medical University, 321 Zhongshan Road, Nanjing, 210008 Jiangsu China; 3 Department of Nuclear Medicine, Nanjing First Hospital, Nanjing Medical University, 68 Changle Road, Nanjing, 210006 Jiangsu China

**Keywords:** Prostate cancer, Multiparametric MRI, ^68^Ga-PSMA-11 PET/CT, T staging, Extracapsular extension

## Abstract

**Background:**

In this study, we explored the diagnostic performances of multiparametric magnetic resonance imaging (mpMRI), ^68^ Ga-PSMA-11 PET/CT and combination of ^68^ Ga-PSMA-11 PET/CT and mpMRI (mpMRI + PET/CT) for extracapsular extension (ECE). Based on the analyses above, we tested the feasibility of using mpMRI + PET/CT results to predict T staging in prostate cancer patients.

**Methods:**

By enrolling 75 patients of prostate cancer with mpMRI and ^68^ Ga-PSMA-11 PET/CT before radical prostatectomy, we analyzed the detection performances of ECE in mpMRI, ^68^ Ga-PSMA-11 PET/CT and mpMRI + PET/CT on their lesion images matched with their pathological sample images layer by layer through receiver operating characteristics (ROC) analysis. By inputting the lesion data into Prostate Imaging Reporting and Data System (PI-RADS), we divided the lesions into different PI-RADS scores. The improvement of detecting ECE was analyzed by net reclassification improvement (NRI). The predictors for T staging were evaluated by using univariate and multivariable analysis. The Kappa test was used to evaluate the prediction ability.

**Results:**

One hundred three regions of lesion were identified from 75 patients. 50 of 103 regions were positive for ECE. The ECE diagnosis AUC of mpMRI + PET/CT is higher than that of mpMRI alone (ΔAUC = 0.101; 95% CI, 0.0148 to 0.1860; *p* < 0.05, respectively). Compared to mpMRI, mpMRI + PET/CT has a significant improvement in detecting ECE in PI-RADS 4–5 (NRI 36.1%, *p* < 0.01). The diagnosis power of mpMRI + PET/CT was an independent predictor for T staging (*p* < 0.001) in logistic regression analysis. In patients with PI-RADS 4–5 lesions, 40 of 46 (87.0%) patients have correct T staging prediction from mpMRI + PET/CT (κ 0.70, *p* < 0.01).

**Conclusion:**

The prediction of T staging in PI-RADS 4–5 prostate cancer patients by mpMRI + PET/CT had a quite good performance.

**Supplementary Information:**

The online version contains supplementary material available at 10.1186/s12894-023-01376-6.

## Background

As prostate cancer has been the fourth most frequent cancer worldwide, there is an urgent need for an accurate primary staging method in order to perform better clinical management [1, 2]. Since ^68^ Ga-PSMA-11 PET/CT and multiparametric magnetic resonance imaging (mpMRI) both have a greater accuracy than conventional imaging, they may have a positive influence on primary staging and patient management for prostate cancer treatment [3, 4]. The combination of ^68^ Ga-PSMA-11 PET/CT and mpMRI (mpMRI + PET/CT) is able to improve the detection of clinically significant prostate cancer (csPCa), which means that more accurate initial diagnosis requires more sophisticated techniques [5].

TNM system (American Joint Committee on Cancer, AJCC) is the most widely used in prostate cancer staging [6]. According to the 8E AJCC, extracapsular extension (ECE) is in T3 [7]. ECE is the adverse risk factor and reference factor of primary staging for prostate cancer, hence their detection plays a vital role in planning surgical strategy and prognosis of patients [8–11]. Among the patients after radical prostatectomy, ECE might be the predictor of biochemical recurrence and shorter survival time [10, 12]. Nowadays, prediction models like nomograms are limited tools without medical imaging information to predict the risk of ECE. It’s urgently needed to use more accurate diagnostic tools to detect ECE so that T staging of prostate cancer can be more accurately detected.

^68^ Ga-PSMA-11 PET/CT is the ^68^ Ga labelled small molecular inhibitor PSMA-11 via the HBED chelator for imaging with positron emission tomography (PET) combined computed tomography (CT) [13, 14]. Nomenclature is in accordance with the International Consensus Radiochemistry Nomenclature Guidelines [15]. Initially, ^68^ Ga-PSMA-11 PET showed favorable sensitivity and specificity in the detection of metastases with biochemical recurrence in prostate cancer [16]. Afterwards, ^68^ Ga-PSMA-11 PET/CT also became the study instrument for primary diagnosis of prostate cancer and performed well [3, 5, 17]. However, the utility of ^68^ Ga-PSMA-11 PET/CT in primary staging and therapy planning of prostate cancer should be evaluated [18].

mpMRI has been reported to be able to mitigate the overdiagnosis or underdiagnosis via mpMRI-targeted biopsy, particularly for the csPCa [19, 20]. Moreover, mpMRI has high specificity for the local staging of prostate cancer including detection of ECE [21]. However, mpMRI has limited sensitivity and is more likely to detect large, solitary, aggressive tumors [21, 22].

This study was aimed at comparing the diagnostic accuracy among ^68^ Ga-PSMA-11 PET/CT, mpMRI and mpMRI + PET/CT for the detection of ECE on a consecutive cohort of patients with whole-mount prostate tissue. At last, the prediction of T staging by mpMRI + PET/CT was presented.

## Methods

### Participants

**Fig. 1:**
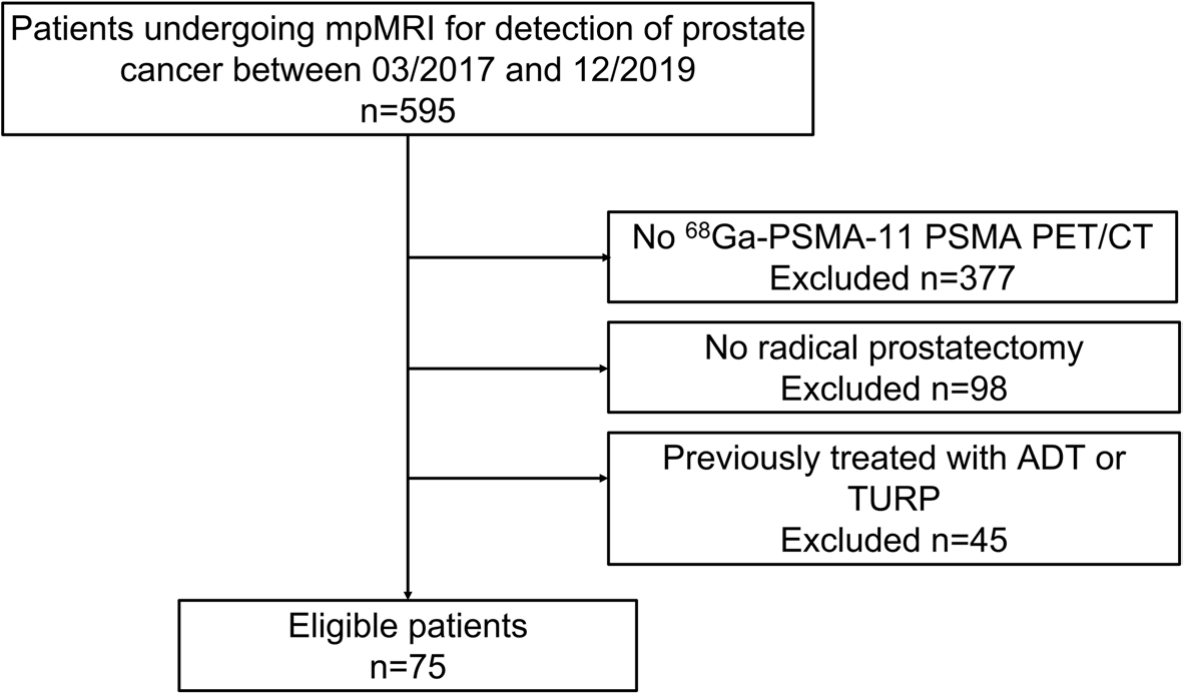
Study Flowchart with excluded patients and reasons for exclusion

A total of 595 consecutive patients of prostate cancer who had undergone 3.0 T mpMRI between March 2017 and December 2019 were retrospectively identified. We excluded the patients with these features as followed: (a) no ^68^ Ga-PSMA-11 PET/CT within 3 months; (b) no radical prostatectomy within 3 months after both ^68^ Ga-PSMA-11 PET/CT and mpMRI; (c) had ADT or TURP before prostatectomy (Fig. 1). There were 75 male patients included in all. The Ethics Committee of the Drum Tower Hospital (2017–147-01) had approved the study and all patients had signed informed consent.

### mpMRI examination

Pelvic mpMRI examinations were performed on patients through a 3.0-T MR scanner (Achieva 3.0 T TX, Philips Medical Systems, The Netherlands) using a 16-channel phased-array coil without endorectal coil [23]. Three planes (Transverse/coronal/sagittal) T2-weighted turbo spin-echo images were obtained. Diffusion-weighted imaging (DWI) spin-echo echo-planar images (b-factor 0/800/1500 s/mm2) were also obtained. Apparent diffusion coefficient (ADC) maps were obtained according to the DWI data. Two dedicated radiologists (15 and 8 years of prostate mpMRI experience) who were blind to ^68^ Ga-PSMA-11 PET/CT and pathologist results, read all the images of mpMRI. Prostate Imaging Reporting and Data System (PI-RADS) Version 2 [24] was used as the reference to score each lesion. A five-point Likert-type scale (where 1 = absent, 2 = probably absent, 3 = equivocal, 4 = probably present, and 5 = definitively present) was used to rate the probability of ECE (each lesion) [25].

### ^68^Ga-PSMA-11 PET/CT examination

The ITG semi-automated module (Munich, Germany) synthesized the ^68^ Ga-PSMA-11 [26]. All patients were intravenously injected with ^68^ Ga-PSMA-11 (median, 131.72 MBq, range 130.6–177.6 MBq) one hour before scanning. The scan machines were a uMI 780 PET-CT scanner (United Imaging Healthcare (UIH), Shanghai, China), a CT scan (130 keV, 80 mAs, slice thickness 3.0 mm) and a static emission scan for correcting dead time, scatter and decay that were obtained from the vertex to the proximal legs (three dimensions matrix 200 × 200). Two dedicated nuclear medicine physicians (13 and 8 years of PET/CT experience) who were blind to mpMRI and pathologist results, read all the images of ^68^ Ga-PSMA-11 PET/CT. A miPSMA expression score (MI-ES) [27] was used as the reference to score each lesion. A five-point Likert-type scale (where 1 = absent, 2 = probably absent, 3 = equivocal, 4 = probably present, and 5 = definitively present) was used to rate the probability of ECE (each lesion) [25].

### Image evaluation

The evaluation of ECE for mpMRI was subjective but guided by the features of PI-RADS, version 2 [24]. The criteria were listed as follows: 1. The recto prostatic angle was obliterated. 2.The interface of the tumor-capsule was greater than 1.0 cm. 3. The tumor extended directly or invaded the bladder wall. 4. The contour of the prostate gland was angulated or spiculate. The evaluation of ECE for ^68^ Ga-PSMA-11 PET/CT was also subjective and the criteria were listed as follows: 1. The accumulation of ^68^ Ga-PSMA-11 was outside of the prostate capsule. 2. The interface of the tumor-capsule was greater than 1.0 cm.3. The rectoprostatic angle was obliterated. 4. The contour of the prostate gland was angulated or spiculate. For evaluation of ECE, the scale of mpMRI + PET/CT was acquired by the scale of mpMRI plus the scale of ^68^ Ga-PSMA-11 PET/CT.

### Whole mount pathological data

According to the Stanford Protocol [28], the whole-mount tissue was first fixed in 10% formalin, and then paraffin embedded. After that, the tissue was microtome cut into 4 mm slices and then stained with hematoxylin–eosin. We scanned the whole mount histology by NanoZoomer Digital Pathology, Shizuoka, Japan. Two dedicated genitourinary pathologists (15 and 9 years of experience) who were blind to mpMRI and.^68^ Ga-PSMA-11 PET/CT results, read all the pathologic images according to the 2014 International Society of Urological Pathology (ISUP) modified criteria for prostate cancer [29].

### Image mark and analysis

According to the slice number, we could match the images of the prostate at the same level. We used green color to draw the border of the prostate. In the images of mpMRI or ^68^ Ga-PSMA-11 PET/CT, we depicted the lesions in blue. As previously stated, the five-point Likert-type scale was subjective but guided by the features mentioned above [25]. In pathologic images, we depicted the lesions in red (Fig. 3). The final histological specimen results were the gold standard for analyzing image results.

### Statistical analysis

All statistical analyses were performed using SPSS Statistics, version 26.0 (IBM Corp., Armonk, NY, USA). The diagnostic performances of ECE on mpMRI, ^68^ Ga-PSMA-11 PET/CT and mpMRI + PET/CT were evaluated according to the receiver operating characteristics (ROC) curves. Area under the curves (AUCs) and 95% CIs were calculated as proposed by Obuchowski [30]. Logistic generalized estimating equation models were used to estimate sensitivities, specificities and CIs [31–33]. A net reclassification improvement (NRI) was used to compare the images with the calculated cutoff [34]. The Kappa test was used to evaluate the prediction ability [35]. The χ2 test was performed for categorical variables. The Mann–Whitney U test was performed for continuous variables. The univariate logistic regression analysis was conducted for all parameters and the multivariate logistic regression analysis was conducted for significant parameters. Two-sided *P* < 0.05 was statistically significant.

## Results

### Patient characteristics

There were 75 patients eligible with the characteristics summarized in Table 1. The median age of the patients was 69 years (range, 55–84 years). The median interval time between mpMRI and radical prostatectomy was 24 days (range 2–57). The median interval time between ^68^ Ga-PSMA-11 PET/CT and radical prostatectomy was 9 days (range 1–79). According to the histologic examination, 48.5% (50 of 103) of the regions were positive for ECE among 64% (48 of 75) of the patients.

**Table 1:** Baseline Features of the Included Cases (*n* = 75) – all underwent mpMRI and ^68^ Ga-PSMA-11 PET/CT and Radical Prostatectomy

Characteristics	Value
Age (years), median (range)	69 (55–84)
Initial PSA (ng/dL), median (range)	14.20 (4.15–120.00)
^a^Gleason score, n (%)	
3 + 3 = 6	4 (5.3)
3 + 4 = 7	27 (36.0)
4 + 3 = 7	22 (29.3)
8 point	11 (14.7)
9–10 point	11 (14.7)
pT stage, n (%)	
2	27 (36.0)
3	48 (64.0)
^a^PI-RADS, n (%)	
3	29 (38.7)
4–5	46 (61.3)
^a^MI-ES, n (%)	
1	7 (9.3)
2	35 (46.7)
3	33 (44.0)
^a^ADCmean (μm2 /s), median (range)	725 (444–1155)
^a^SUVmax, median (range)	14.23 (5.54–90.34)
Percentage of positive core at TB (%), median(range)	36 (7–100)
ISUP Gleason score at TB, n (%)	
1	13 (17.3)
2	14 (18.7)
3	9 (12.0)
4	27 (36.0)
5	12 (16.0)

*mpMRI* multiparametric magnetic resonance imaging, *PET/CT* positron emission tomography computed tomography, *PSA* prostate-specific antigen, *ISUP* International Society of Urological Pathology, *TB* targeted biopsy, *PI-RADS* Prostate Imaging Reporting and Data System, *MI-ES* a miPSMA expression score, *ADCmean* average apparent diffusion coefficient, *SUVmax* maximum standardized uptake value

^a^For patients with multifoci, the data are based on index lesions

### Diagnostic performance for the detection of ECE

**Fig. 2:**
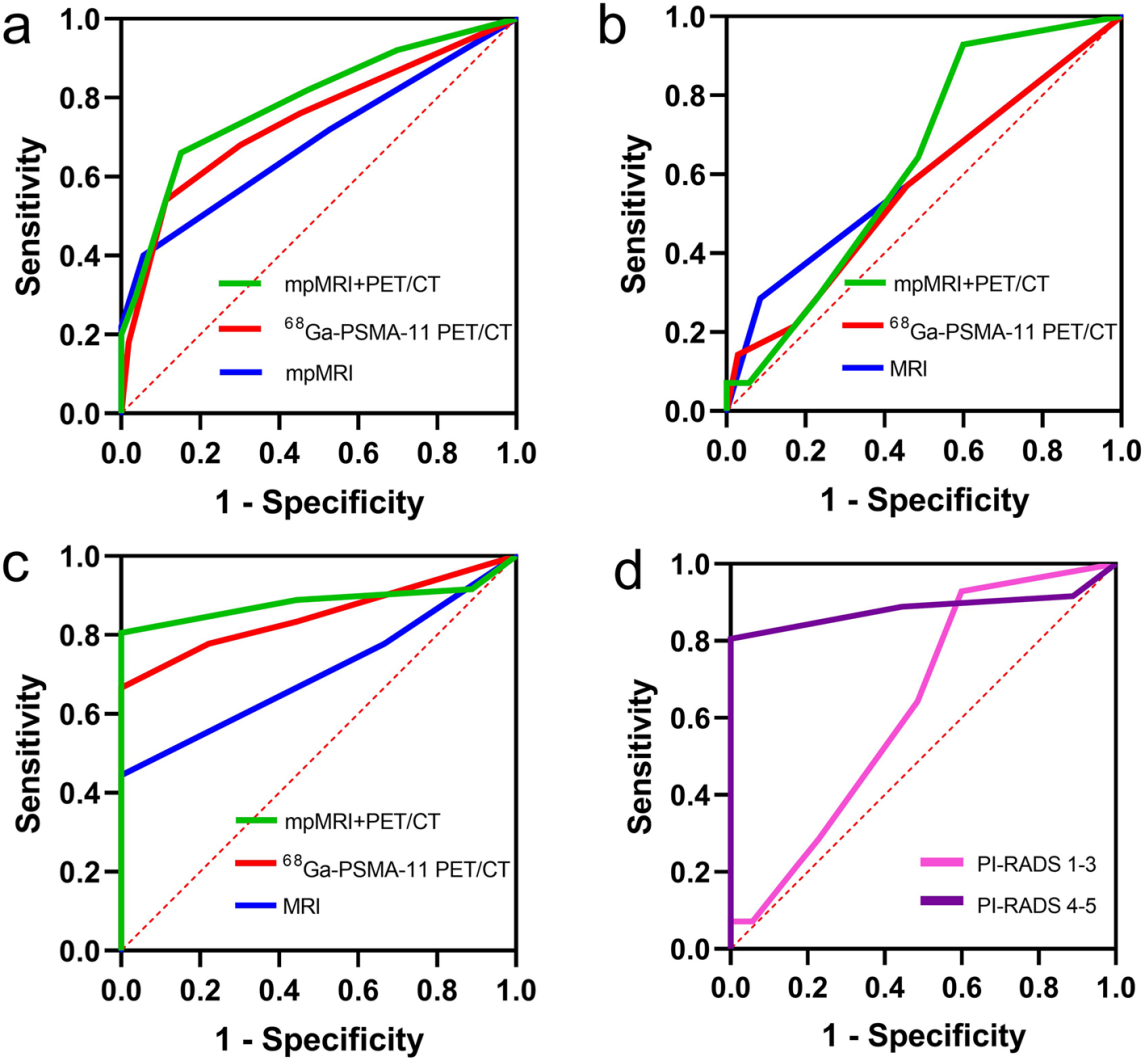
Receiver operating characteristics (ROC) analyses. **a** ROC analyses of mpMRI, ^68^ Ga-PSMA-11 PET/CT and mpMRI + PET/CT for the detection of ECE of all the lesions; **b** ROC analyses of mpMRI, ^68^ Ga-PSMA-11 PET/CT and mpMRI + PET/CT for the detection of ECE of lesions PI-RADS 1–3; **c** ROC analyses of mpMRI, ^68^ Ga-PSMA-11 PET/CT and mpMRI + PET/CT for the detection of ECE of lesions PI-RADS 4–5; **d** ROC analyses of lesions PI-RADS 1–3 and PI-RADS 4–5 for the detection of ECE by mpMRI + PET/CT

The ROC curves of ECE region-specific analyses are illustrated in Fig. 2 with AUC and 95% CI for each image shown in Table 2. The AUC of mpMRI + PET/CT improved ECE diagnosis comparing to that of mpMRI alone (ΔAUC = 0.101; 95% CI, 0.0148 to 0.1860; *p* < 0.05). However, there was no significant difference in AUC between mpMRI + PET/CT and ^68^ Ga-PSMA-11 PET/CT (ΔAUC = 0.047; 95% CI, -0.0052 to 0.0992; *p* = 0.08). Besides, there was no significant difference in AUC between mpMRI and ^68^ Ga-PSMA-11 PET/CT (ΔAUC = 0.053; 95% CI, -0.0690 to 0.1760; *p* = 0.39).

**Table 2:** Diagnostic Accuracies for ECE Using mpMRI, ^68^ Ga-PSMA-11 PET/CT and MRI + PET/CT compared to Final Histology

		mpMRI	^68^ Ga-PSMA-11 PET/CT	mpMRI + PET/CT
PI-RADS 1–5				
AUC (95%CI)		0.69 (0.58–0.79)*	0.74 (0.64–0.84)	0.79 (0.70–0.88)*
Youden-selected threshold		3	4	5
Sensitivity, %, at threshold (95%CI)		40 (28–54)*	54 (40–67)†	66 (52–78) *†
Specificity, %, at threshold (95%CI)		94 (85–98)	89 (77–95)	85 (73–92)
ECE	Positive	20	27	33
	Negative	30	23	17
None-ECE	Positive	3	6	8
	Negative	50	47	45
PI-RADS 1–3				
AUC (95%CI)		0.60 (0.41–0.78)	0.56 (0.38–0.74)	0.62 (0.47–0.79)
Youden-selected threshold		3	3	3
Sensitivity, %, at threshold (95%CI)		28 (12–55)	57 (32–79)	92 (68–100)
Specificity, %, at threshold (95%CI)		91 (78–97)	54 (38–70)	40 (26–56)
ECE	Positive	4	6	13
	Negative	10	8	1
None-ECE	Positive	3	12	21
	Negative	32	23	14
PI-RADS 4–5				
AUC (95%CI)		0.70 (0.57–0.84)*	0.85 (0.75–0.95)	0.88 (0.79–0.98)*
Youden-selected threshold		3	4	5
Sensitivity, %, at threshold (95%CI)		44 (30–60)*	66 (50–80)	80 (65–90)*
Specificity, %, at threshold (95%CI)		100 (82–100)	100 (82–100)	100 (82–100)
ECE	Positive	16	24	29
	Negative	20	12	7
None-ECE	Positive	0	0	0
	Negative	18	18	18

*PI-RADS* Prostate Imaging Reporting and Data System, *AUC* area under the curve, *CI* confidence interval, *mpMRI* multiparametric magnetic resonance imaging, *PET/CT* positron emission tomography computed tomography, *ECE* extracapsular extension, *mpMRI* + *PET/CT* combination of ^68^ Ga-PSMA-11 PET/CT and mpMRI

^*^ mpMRI versus mpMRI + PET/CT, *p* < 0.05

^†^ PET/CT versus mpMRI + PET/CT, *p* < 0.05

Table 2 also shows the Youden-selected threshold, sensitivity and specificity for each image. The cutoff of mpMRI, ^68^ Ga-PSMA-11 PET/CT and mpMRI + PET/CT calculated by the Youden-selected threshold and the actual diagnostic results are listed in Table 2. The sensitivity of mpMRI + PET/CT was higher both than mpMRI and ^68^ Ga-PSMA-11 PET/CT(*p* < 0.05 and *p* < 0.05), with no sacrifice on specificity (*p* = 0.34 and *p* = 0.50). There was no significant difference in sensitivity and specificity between mpMRI and ^68^ Ga-PSMA-11 PET/CT (*p* = 0.17 and *p* = 0.72). Compared with mpMRI, mpMRI + PET/CT had a positive NRI (NRI 16.6%, *P* = 0.051) with the calculated cutoff (Supplemental Table 1).

Dividing the lesions into PI-RADS 1–3 and PI-RADS 4–5, the ROC curves and AUC of ECE (95% CI) are shown in Fig. 2 and Table 2. In the group of PI-RADS 1–3, there was no significant difference in AUC between mpMRI and ^68^ Ga-PSMA-11 PET/CT, mpMRI + PET/CT and mpMRI, mpMRI + PET/CT and ^68^ Ga-PSMA-11 PET/CT (ΔAUC = 0.032; 95% CI, -0.2360 to 0.2990; *p* = 0.82, ΔAUC = 0.032; 95% CI, -0.1510 to 0.2140; *p* = 0.73, ΔAUC = 0.063; 95% CI, -0.0432 to 0.1700; *p* = 0.24). In the group of PI-RADS 4–5, the AUC of mpMRI + PET/CT improved ECE diagnosis compared to that of mpMRI alone (ΔAUC = 0.181; 95% CI, 0.0660 to 0.2950; *p* < 0.01). However, there was no significant difference for AUC between mpMRI and ^68^ Ga-PSMA-11 PET/CT, mpMRI + PET/CT and ^68^ Ga-PSMA-11 PET/CT (ΔAUC = 0.145; 95% CI, -0.0041 to 0.2940; *p* = 0.06, ΔAUC = 0.036; 95% CI, -0.0329 to 0.1040; *p* = 0.31). For further exploration, the NRIs were analyzed and listed in Supplemental Table 1. Compared to mpMRI, mpMRI + PET/CT showed that lesions with PI-RADS 4–5 had a significant improvement (36.1%, *p* < 0.001) while lesions with PI-RADS 1–3 had no significant improvement (12.9%, *p* = 0.223). The sensitivity of mpMRI + PET/CT was higher than mpMRI in lesions with PI-RADS 4–5 (*p* < 0.05) (Table 2).

**Fig. 3:**
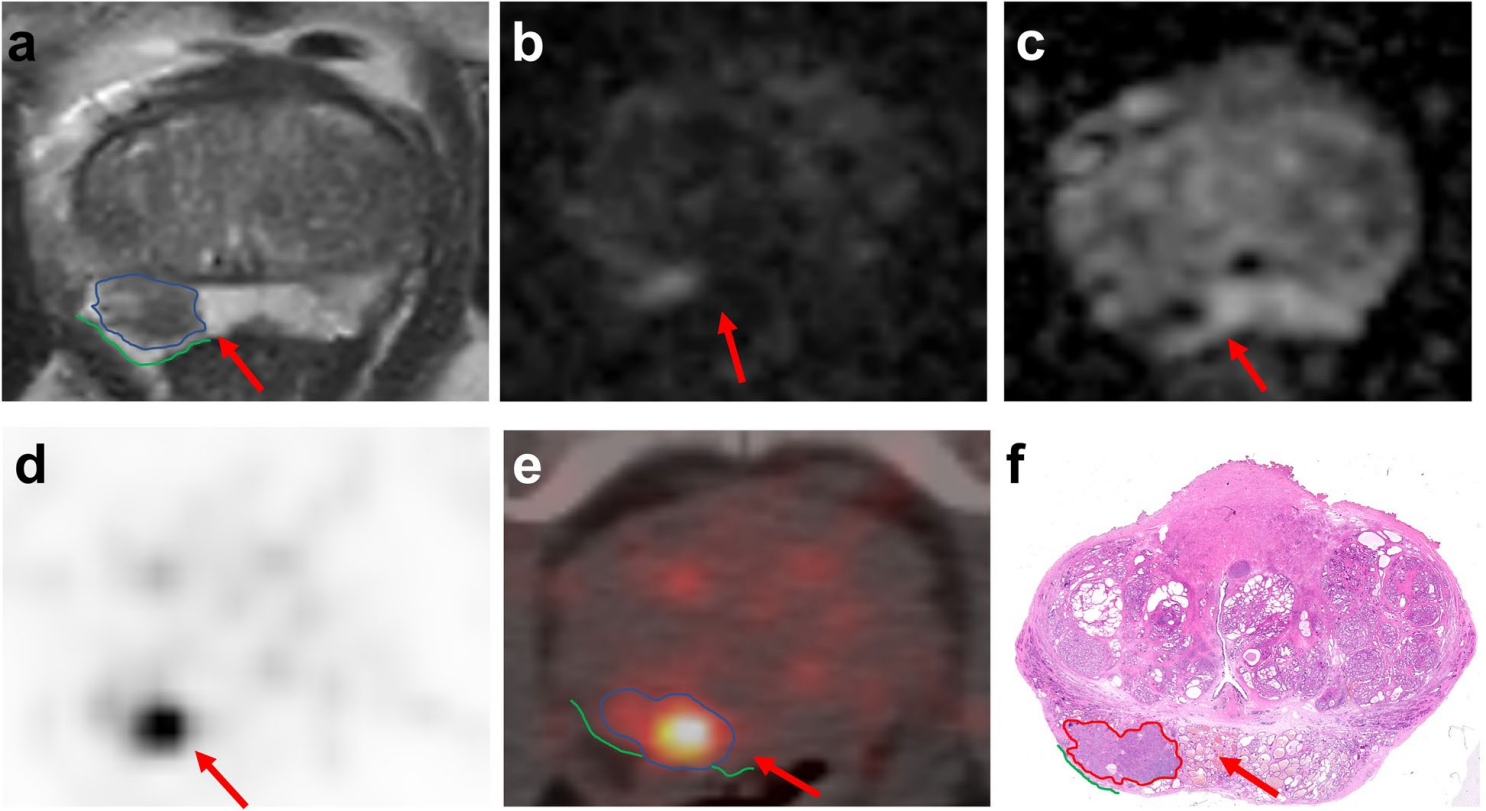
The images of a 67-year-old patient with prostate-specific antigen of 5.46 ng/ml. **a** Transverse T2-weighted images on MRI showed a lesion in the right peripheral zone (red arrow). **b** DWI with b1500 shows a moderately high signal on the edge of right peripheral zone (red arrow). **c** ADC map showed a moderate hypo intensity on the edge of right peripheral zone (red arrow). All the finding results in a PI-RADS 3. **d, e** PET showed great intense focal uptake on the right peripheral zone (red arrow), which is equal to the parotid gland, resulting in an MI-ES 3. Readers rated the images from mpMRI as negative for extraprostatic extension (Likert scale points = 2), whereas they rated the images from.^68^ Ga-PSMA-11 PET/CT and mpMRI + PET/CT as positive for extraprostatic extension (Likert scale points = 4 and 6). **f** Whole mount histology confirms the tumor in the right peripheral zone without extraprostatic extension (red arrow)

Figure 3 shows examples of mpMRI and ^68^ Ga-PSMA-11 PET/CT results. Figure 3 shows a case of false-positive ECE mpMRI + PET/CT with PI-RADS 3.

### Univariable and multivariable logistic regression for prediction of T staging

Table 3 showed that compared to patients in T2, patients in T3 had a higher initial prostate-specific antigen (PSA), a larger percentage of the positive core at targeted biopsy (TB), higher ISUP Gleason score at TB and a higher scale in the evaluation of ECE by mpMRI + PET/CT. Table 4 showed the results of univariable and multivariable logistic regression for the prediction of T staging. In univariable analysis, initial PSA, percentage of positive core at TB, ISUP Gleason score at TB and mpMRI + PET/CT were significantly associated with T staging. Multivariable logistic regression analysis demonstrated that only mpMRI + PET/CT (OR = 11.337; 95% CI: 3.088 – 41.916; *p* < 0.001) was an independent predictor of T staging.

**Table 3:** Clinical and imaging characteristics of patients (*n* = 75) with T2 and T3 in final pathology

	T2 (27, 36.0%)	T3 (48, 64.0%)	*p*
Age (years), median (range)	69 (58–82)	68 (55–84)	0.868
Initial PSA (ng/dL), median (range)	9.87 (4.15–60.17)	15.06 (5.42–120)	**0.016**
Percentage of positive core at TB (%), median (range)	28.6 (7.1–81.2)	41.7 (7.1–100)	**0.031**
ISUP Gleason score at TB, n (%)			**0.032**
1	8 (29.6)	5 (10.4)	
2	8 (29.6)	6 (12.5)	
3	3 (11.1)	6 (12.5)	
4	6 (22.2)	21 (43.8)	
5	2 (7.4)	10 (20.8)	
mpMRI + PET/CT, n (%)			
scale			**0.013**
2	1 (3.7)	3 (6.3)	
3	9 (33.3)	6 (12.5)	
4	12 (44.4)	7 (14.6)	
5	1 (3.7)	6 (12.5)	
6	3 (11.1)	12 (25.0)	
7	1 (3.7)	4 (8.3)	
8	0 (0)	3 (6.25)	
9	0 (0)	5 (10.4)	
10	0 (0)	2 (4.16)	
Diagnosis			** < 0.001**
Positive	5	32	
Negative	22	16	

*PSA* prostate-specific antigen, *ISUP* International Society of Urological Pathology, *TB* targeted biopsy, *mpMRI* + *PET/CT* combination of ^68^ Ga-PSMA-11 PET/CT and mpMRI

Significant *P* values were presented in bold text

**Table 4:** Univariable and multivariable logistic regression analysis of possible predictors for distinguishing patients (*n* = 75) between T2 and T3 in final pathology

Parameters	Univariable logistic regression			Multivariable logistic regression		
	OR	95% CI	*p*	OR	95% CI	*p*
Age (years), median (range)	1.000	0.928 – 1.079	0.990			
Initial PSA (ng/dL), median (range)	1.029	1.001 – 1.058	**0.042**	1.027	0.992 – 1.064	0.136
Percentage of positive core at TB (%), median (range)	1.029	1.001 – 1.057	**0.041**	0.637	0.972 – 1.048	0.637
ISUP Gleason score at TB, n (%)			**0.044**			0.234
1 vs. 2	1.200	0.257 – 5.593	0.816	0.759	0.105 – 5.473	0.784
1 vs. 3	3.200	0.540 – 18.980	0.200	2.168	0.250 – 18.833	0.483
1 vs. 4	5.600	1.328 – 23.620	**0.019**	2.755	0.425 – 17.868	0.288
1 vs. 5	8.000	1.215 – 52.693	**0.031**	9.842	0.789 – 122.734	0.076
mpMRI + PET/CT						
Negative vs. Positive	8.800	2.810 – 27.557	** < 0.001**	11.377	3.088 – 41.916	** < 0.001**

*PSA* prostate-specific antigen, *ISUP* International Society of Urological Pathology, *TB* targeted biopsy, *mpMRI* + *PET/CT* combination of ^68^ Ga-PSMA-11 PET/CT and mpMRI, *OR* odds ratio, *CI* confidence intervals

Significant *P* values were presented in bold text

###  Prediction of T staging by mpMRI + PET/CT

Because mpMRI + PET/CT had better performance in patients with PI-RADS 4–5 lesions, we used mpMRI + PET/CT to predict T staging. There were 46 patients enrolled. The cutoffs of ECE were 5. 40 of 46 (87.0%) patients have correct prediction. The κ statistic was 0.70, *p* < 0.01, which indicated a fair consistency (Supplemental Table 2).

## Discussion

To our knowledge, this study is the first to use mpMRI + PET/CT to predict the T staging in prostate cancer patients. The role of mpMRI + PET/CT as an independent predictor of T staging has never been demonstrated before. Based on the mpMRI + PET/CT’s improvement in the detection of ECE compared to mpMRI, especially in PI-RADS 4–5, we found that T staging might have the considerable consistency between mpMRI + PET/CT and final pathology. By using mpMRI + PET/CT for primary detection of prostate cancer, we can determine the mode and scope of surgery according to the predicted results, which is conducive to clinical work [17].

In our study, we wanted to further explore the relationship between ^68^ Ga-PSMA-11 PET/CT and final pathology at first. However, ^68^ Ga-PSMA-11 PET/CT also had some disabilities like image fusion deviation or differences in concentration, action time, individual metabolic of tracers and etc. To fill these gaps, we included mpMRI and clinical features to improve the predictive power for final pathology [5]. Through a series of analyses, we found that mpMRI + PET/CT had a fair performance in the prediction of T staging.

For economic considerations, mpMRI + PET/CT may be better than ^68^ Ga-PSMA-11 PET/MRI. For patients with PI-RADS 1–3, ^68^ Ga-PSMA-11 PET/CT or PET/MRI is not a prerequisite for early staging [25]. Although mpMRI + PET/CT can improve the detection of csPCa for lesions with PI-RADS 3, we should still adopt a prudent policy in patients with lesions with PI-RADS 3, especially when the ECE is positive for ^68^ Ga-PSMA-11 PET/CT but negative for mpMRI [5]. Compared to ^68^ Ga-PSMA-11 PET/CT, mpMRI costs less and has less damage to human health. We chose the right time to use the proper imaging tools, so that they can have the maximum value in clinical diagnosis. Beyond that, patients’ suffering and medical expenses should be reduced for humanitarian reasons.

For more accurate prediction of T staging, mpMRI + PET/CT should be included in the Nomogram with more clinical features for prediction of ECE [36, 37]. With the improvement of prediction ability with comprehensive clinical information, miTNM will play a greater value in prostate cancer diagnosis [27]. Nowadays, it still lacks favorable evidence that ^68^ Ga-PSMA-11 PET/MRI has better performance than ^68^ Ga-PSMA-11 PET/CT. ^68^ Ga-PSMA-11 PET/MRI has a long inspection time and higher cost. Except for that, most patients would conduct mpMRI before ^68^ Ga-PSMA-11 PET/CT or ^68^ Ga-PSMA-11 PET/MRI, which means that ^68^ Ga-PSMA-11 PET/MRI would have a certain degree of repeated inspection.

As highly sensitive imaging diagnostic tools, the sensitivity and specificity of ^68^ Ga-PSMA-11 PET/CT were, respectively, 90.0% and 90.9% for ECE [38]. Besides, ^68^ Ga-PSMA-11 PET/MRI had an increased sensitivity for ECE compared to mpMRI (69% vs 46%, *p* = 0.04) [25]. In our study, the mpMRI + PET/CT also had higher sensitivity in ECE than mpMRI (66% vs 40%, *p* < 0.05). However, specificity had no increase or even slight reduction, especially in PI-RADS 3. It is likely due to an image fusion error. In future research, we want to find the influence factors of specificity reduction in ^68^ Ga-PSMA-11 PET/CT or mpMRI + PET/CT.

^68^ Ga-PSMA-11 PET/CT has the potential advantage in prostate cancer primary staging and the combination of mpMRI had higher accuracy [39]. In our study, we combined mpMRI and ^68^ Ga-PSMA-11 PET/CT to predict T staging in PI-RADS 4–5 (87.0%, 0.70, *p* < 0.01). The result demonstrated that we could have more accurate local staging prior to surgery.

The limitations of our study are listed as follows: 1. Selection bias: all patients underwent surgery in order to obtain pathological specimens, hence, no negative or low risk patients were included. 2.Limited samples: our study enrolled 75 patients which might have a certain influence on the reliability of results. But for a variety of reasons, there were only a handful of patients who met all the requirements.3. Heterogeneity: because of this study’s retrospective nature, there were many variables not under full control, including but not limited to the examination duration and the examination intervals.

## Conclusions

Our study verifies that mpMRI + PET/CT can improve ECE diagnosis compared to mpMRI, especially in prostate cancer patients with PI-RADS 4–5 lesions. The diagnosis of mpMRI + PET/CT was an independent predictor (*p* < 0.001) in logistic regression analysis. The prediction power of T staging in PI-RADS 4–5 prostate cancer by mpMRI + PET/CT was moderate. These results may help clinical decisions on primary staging for prostate cancer diagnosis before surgical operation in a more economical way.

### Supplementary Information

**Additional file 1: Supplemental Table 1. **Diagnostic Changement for the Detection of ECE by mpMRI+PET/CT compared to mpMRI.

**Additional file 2: Supplemental Table 2. **Prediction Consistency of T stage in PI-RADS 4-5 Prostate Cancer by mpMRI+PET/CT.

## Data Availability

The datasets used and/or analyzed during the current study are available from the corresponding author upon reasonable request.

## Abbreviations

mpMRI: Multiparametric magnetic resonance imaging
mpMRI + PET/CT: Combination of ^68^ Ga-PSMA-11 PET/CT and mpMRI
csPCa: Clinically significant prostate cancer
AJCC: American Joint Committee on Cancer
ECE: Extracapsular extension
PET: Positron emission tomography
CT: Computed tomography
DWI: Diffusion-weighted imaging
ADC: Apparent diffusion coefficient
ISUP: International Society of Urological Pathology
PI-RADS: Prostate Imaging Reporting and Data System
MI-ES: MiPSMA expression score
ROC: Receiver operating characteristics
AUC: Area under the curve
NRI: Net reclassification improvement
PSA: Prostate-specific antigen
TB: Targeted biopsy
