# Supplementary material for: Prediction of T staging in PI-RADS 4–5 prostate cancer by combination of multiparametric MRI and 68Ga-PSMA-11 PET/CT
Source: BMC Urol. 2023 Dec 11;23:206. doi: 10.1186/s12894-023-01376-6 (PMC10712094; doi:10.1186/s12894-023-01376-6)
Supplement: Supplementary file 1 — Additional file 1: Supplemental Table 1. Diagnostic Changement for the Detection of ECE by mpMRI+PET/CT compared to mpMRI. [file 12894_2023_1376_MOESM1_ESM.docx]

**Supplemental TABLE 1** Diagnostic Changement for the Detection of ECE by mpMRI+PET/CT compared to mpMRI.

|  | NRI, % | *p* |
| --- | --- | --- |
| PI-RADS 1-5 | 16.6 | 0.051 |
| PI-RADS 1-3 | 12.9 | 0.223 |
| PI-RADS 4-5 | 36.1 | **<0.001** |

*mpMRI* multiparametric magnetic resonance imaging, *mpMRI+PET/CT* combination of ^68^Ga-PSMA-11 PET/CT and mpMRI *NRI* Net Reclassification Index, *PI-RADS* Prostate Imaging Reporting and Data System

Significant *P* values were presented in bold text
