# Supplementary material for: Prediction of T staging in PI-RADS 4–5 prostate cancer by combination of multiparametric MRI and 68Ga-PSMA-11 PET/CT
Source: BMC Urol. 2023 Dec 11;23:206. doi: 10.1186/s12894-023-01376-6 (PMC10712094; doi:10.1186/s12894-023-01376-6)
Supplement: Supplementary file 2 — Additional file 2: Supplemental Table 2. Prediction Consistency of T stage in PI-RADS 4-5 Prostate Cancer by mpMRI+PET/CT. [file 12894_2023_1376_MOESM2_ESM.docx]

**Supplemental TABLE 2** Prediction Consistency of T stage in PI-RADS 4-5 Prostate Cancer by mpMRI+PET/CT

|  |  | Pathology | |
| --- | --- | --- | --- |
|  |  | T2 | T3 |
| mpMRI+PET/CT | T2 | 11 | 7 |
|  | T3 | 0 | 28 |
| κ | 0.70 | p | **<0.001** |

*mpMRI* multiparametric magnetic resonance imaging, *mpMRI+PET/CT* combination of ^68^Ga-PSMA-11 PET/CT and mpMRI

Significant *P* values were presented in bold text
